# Supplementary material for: scafSLICR: A MATLAB-based slicing algorithm to enable 3D-printing of tissue engineering scaffolds with heterogeneous porous microarchitecture
Source: PLoS One. 2019 Nov 19;14(11):e0225007. doi: 10.1371/journal.pone.0225007 (PMC6863524; doi:10.1371/journal.pone.0225007)
Supplement: S1 Appendix — (DOCX) [file pone.0225007.s001.docx]

scafSLICR: a MATLAB-based Slicing Algorithm to Enable Fused Deposition Modeling 3D-Printing of Tissue Engineering Scaffolds with Heterogenous Porous Microarchitecture

Ethan Nyberg & Aine O'Sullivan, Warren Grayson

# Appendix A

**Files available at:**

<https://www.dropbox.com/sh/dr7j8i8y0sbxo8t/AACYmzJ0irz9B2s1q9Z43Ulva?dl=0>

### Overview

This program takes 3D-shapes as an input and applies patterns of pores and struts to different regions. It then generates the GCODE necessary to manufacture that structure.

### Directory Set Up

The *scafSLICR.mlapp*  file should be in the current MATLAB directory. The subdirectories *sub functions*, *Shapes*, *Properties*, *Output*, and *gcode* should also be in the current directory.

- ***sub functions*** contains the sub-functions and scripts needed.
- ***Shapes*** contains some example shapes and the shapes used in these examples
- ***Output*** is used to pass data from the slicing function back to the GUI, and where the final output gcode file is deposited.
- ***gcode*** contains the gcode header and footer files applied to the gcode.

Initialize the program with the **scafSLICR** command in command window, not by opening the mlapp files.


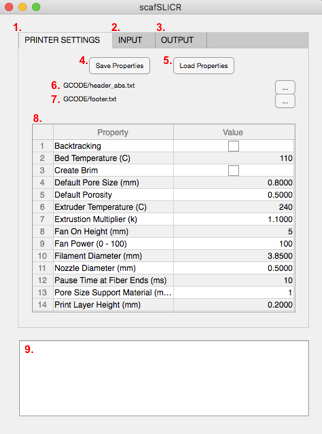


### Program Tabs

1. **Printer Settings** This tab contains all the settings needed to alter the printer (hardware) and material-specific settings of the slicing algorithm.
2. **Input**. This tab contains the controls to input a shape and manipulate the porous properties throughout the shape.
3. **Output.** This tab contains toolpath previews of the sliced design.

### Properties Buttons

1. **Save Properties.** This button saves the printer and material settings to a file to re-use.
2. **Load Properties.** This button loads previously saved printer settings.

GCODE Header / Footer. The GCODE header and footer files are identified. These can be edited directly in the text files using standard programs (TextEdit or Notepad). The **…** buttons can be used to select different text files to use as the header and footer.

1. **Header.** The GCODE header contains machine instructions to prepare the printer to manufacture the print. This text file is appended to the front of the shape-specific GCODE instructions.
2. **Footer.** Similar to the header, these instructions are appended after the shape-specific print is completed and might move the extruder head out of the way, kick the bed out, and set the temperatures to cool off.

### Printer Settings Table (8)

The Printer Property table contains the machine and material specific settings. See the table in the slicerFN section for explanation of each property.

### Output Console (9)

The output console is in the lower panel of the program. Status updates and errors are displayed here. The text can be edited to include user notes. This console is for output only, and it does not take any inputs.


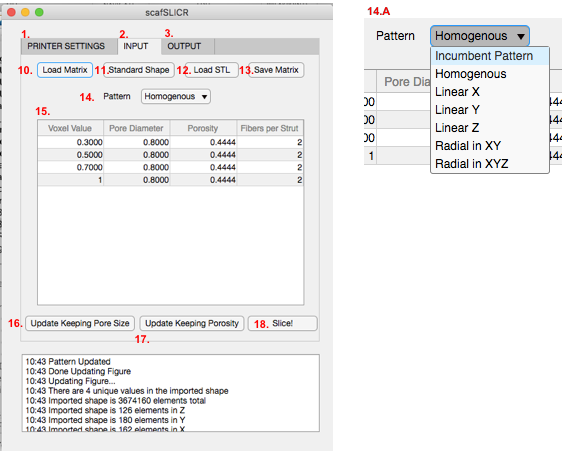


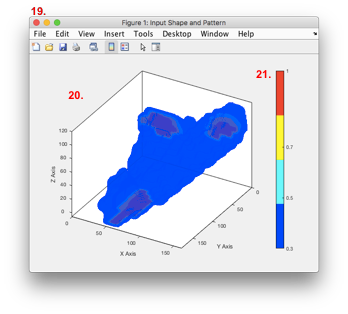


### Load A Shape

10. Load Matrix: Import a matrix (*x* x *y* x *z*). The values of each element / voxel can be used to pattern a design value in the shape. The scale of a voxel in the imported model is set on the printer settings table.

11. Standard Shape: Click through the resulting dialog boxes to generate a cylinder or rectangular standard shape.

**12. Load STL**: Import an STL. This process may take a minute or two depending on the size of the STL. It is assumed that the STL is scaled in millimeters.

After the shape is loaded and patterned, it can be saved it as a matrix to re-use or manipulate further. Because loading matrices is faster than creating a standard shape or loading an STL, this format can speed up recurring designs. Matrices can be exported as to use in other modeling programs.

19. Input Shape Window. This window shows the loaded shape (20) where the different colors (Legend 21) correspond to the voxel values in the table (15).

**Pattern Options (14A).** Clicking a new pattern option from the dropdown menu will change the input shape and pore properties table to match that pattern. Some patterns require additional input via dialog boxes.

- **Incumbent Pattern**
- **Homogenous**
- **Linear X**
- **Linear Y**
- **Linear Z**
- **Radial in XY**
- **Radial in XYZ**

**15. Porous Properties.** This table displays the different regions of the shape (voxel values) and can be edited to assign different pore size and porosity to each region. This screenshot shows the same porous pattern applied to the different regions to result in a homogenous scaffold.

**16. & 17. Update Buttons.** Because there are only certain manufacturable combinations of struts and pores, the update buttons below the table will fit the input pore size / porosity to the nearest manufacturable set.

18. Slice. This button starts the slicing algorithm. Check all the inputs (printer settings, shape, and pore table) before clicking it.


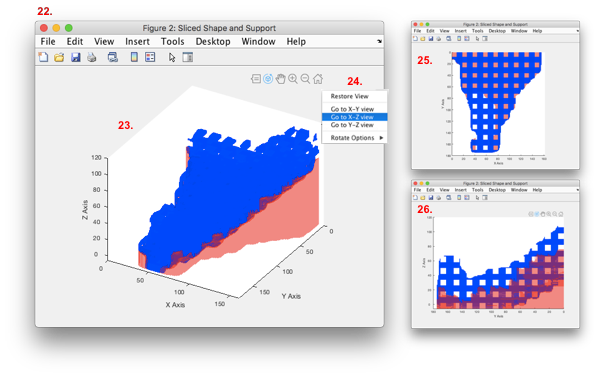


**Sliced Shape and Support Window (22).** This window displays the sliced shape and its support (23). Blue is the print structure and red is support structure. Right-click (24) gives the view options. X-Y view (25) and Y-Z view (26) both show the pores throughout the structure.


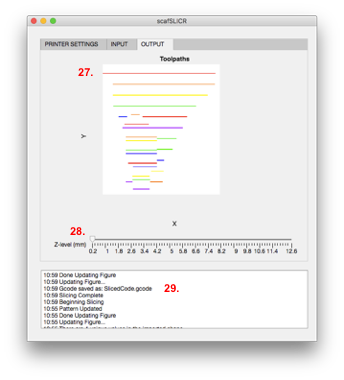


**27. 2D Toolpath Previews**. The tool paths are shown in the third tab. Each tool-path is a separate color. The different layers can be viewed by moving the scroll bar (28) through the different z-levels.

The generated GCODE is saved in the *output* folder as 'SlicedCode.gcode' (29), and it can be opened in a program such as Slicr or Repetier to ensure that the toolpaths are as desired in 3D.

**Using SlicrFn as MATLAB Function Directly**

The slicing function can be implemented programmatically in MATLAB instead of through the GUI.

Syntax

SlicrFn(V, VoxelSize, PoreDiameter, Porosity, Position)

SlicrFn(___, Name, Value)

[gcode, toolPaths, preview] = SlicrFn(___)

Description

V A 3D-volume matrix describing the shape to be sliced. Each unique value in V corresponds to a pore/porosity design choice.

VoxelSize The length of a voxel edge in mm. e.g. 0.100

PoreDiameter A matrix corresponding to the ordered unique values of V, where each element is the pore size for that design choice. Provided in mm. e.g. [ 0.800 0.400 1.200 ]

Porosity A matrix corresponding to the ordered unique values of V, where each element is the porosity for that design choice. Provided in fractions. e.g. [ 0.90 0.45 0.23 ]

Position [ x_pos y_pos ] The initial x and y position of the printed scaffold on the print bed. The program places the origin of the scaffold at this location.

Name, Value pairs provide additional inputs to the slicing program.

| **Name** | **Value** | **Description** |
| --- | --- | --- |
| Backtracking | Boolean | Option to backtrack along the last portion of the toolpath so that oozing and drawn strands do not close off the side pores. Default = true. |
| BedTemp | Celsius | The temperature of the bed during printing. Degrees Celsius. Default = 100 |
| CreateBrim | Boolean | Option to create a brim around the perimeter of the object on the print bed. Default = true. |
| ExtruderTemp |  | the heat setting of the nozzle during printing. In degrees Celsius. default = 285. If multiple extruders, provide as [ temp1 temp2]. |
| ExtrusionMultiplier | 0.8 – 1.5 | increases the material flow rate to account for over / under deposition of material. A value of 1 would result in a perfect flow rate. Slight over deposition 1.05 is generally good. Default = 1.05 |
| FanHeight | mm | z-height at which the fan turns on. Default = 1mm. |
| FanPower | 0 – 100 | Intensity of the fan, when it is turned on. Default = 100. |
| FilamentDiameter | mm | The diameter of the filament used. Default – 2.85mm. If using multiple extruders, provide as [diam1 diam2]. |
| LayerHeight | mm | the z-change for each print layer. default = 0.200mm |
| NozzleDiameter | mm | the diameter of the primary printer nozzle, and determines the width of a strand. Struts can be integer multiples of the strand width (1, 2, 3 times the nozzle width.) default = 0.500mm. If multiple extruders provide as [diam1 diam2] |
| PauseTime | ms | Option to pause at the end of each toolpath to let it cool before starting the next move. Default = 50. If 0, no pause time. |
| PrintSpeed | mm/min | the speed of the nozzle. mm/min. default = 90. |
| SupportPoreDiam | mm | The spacing between the support struts. Default = 1mm. |
|  |  |  |

Outputs:

By default, SlicerFn creates the outputs in the Output directory, and it saves three files:

- preview.mat: contains the 3D-matrix Tt and the 1D-matrix Zz. Tt(:,:,Zz(i)) is the toolpaths selected for the ith print layer. Preview with imshow( Tt(:,:,Zz(i)) ).
- Graphic_Matrix.mat: contains the 3D-matrices of the scaffold and support. Inspect with plot_3d
- glines.mat: contains a string array of the different lines of gcode, which is ready to be packaged into a text file with start up and shut down machine specific gcode.

If outputs are specified:

gcode An array of the gcode commands. It does not include header / footer gcode, and should be compiled with those to create useful gcode files.

toolPaths A structured output with two parts:

.z_heights is an array where each element is one of the slices and indicates the z-height of that slice

.ToolPaths is a 3D-matrix of the tool paths for each print layer. The nth member of .z_heights is height of ToolPaths.ToolPaths(:,:,n)

preview A structured output with two parts:

.scaffold is the graphic preview of the sliced scaffold

.support is the graphical preview of the support structure

Example

load('Shapes/zygoma.mat')

V = A;

VoxelSize = 0.100;

PoreDiameter = [ 0.3 0.5 0.7 1.0 ];

Porosity = [ 0.375 0.5 0.4118 0.500 ];

% Porosity and PoreDiameter must be the same length as the number of

% non-zero unique values in V, and are ordered respectively to the

% non-zero output of unique(V).

position = [15 15];

[glines, toolPaths, preview] = SlicrFn ( V, ...

VoxelSize, ...

PoreDiameter, ...

Porosity, ...

position,...

'Backtracking', false,...

'BedTemp', 110,...

'CreateBrim', true,...

'ExtruderTemp', 285,...

'ExtrusionMultiplier', 1.1,...

'FanHeight', 1,...

'FanPower', 95,...

'FilamentDiameter', 2.85,...

'LayerHeight', 0.200,...

'NozzleDiameter', 0.500,...

'PauseTime', 200,...

'PrintSpeed', 600,...

'SupportPoreDiam', 0.95...

);

%% Graph the output preview

figure, p = plot_3d(preview.scaffold,0.1, 1);

%% Complete the GCODE as file

% Get GCODE Header and footer files

fileID = fopen(fullfile('gcode','header_abs.txt'),'r');

gstart = textscan(fileID,'%s','delimiter','\n');

gstart = string(gstart{1});

fclose(fileID);

fileID = fopen(fullfile('gcode','footer.txt'),'r');

gend = textscan(fileID,'%s','delimiter','\n');

gend = string(gend{1});

fclose(fileID);

% Read GCODE lines into output file

fileID = fopen(fullfile('gcode','SlicedCode.gcode'),'w');

formatSpec = '%s\n';

fprintf(fileID,formatSpec,gstart);

glines(glines == '')=[]; %remove empty dimensions

fprintf(fileID,formatSpec,glines');

fprintf(fileID,formatSpec,gend);

fclose(fileID);
