# Supplementary material for: scafSLICR: A MATLAB-based slicing algorithm to enable 3D-printing of tissue engineering scaffolds with heterogeneous porous microarchitecture
Source: PLoS One. 2019 Nov 19;14(11):e0225007. doi: 10.1371/journal.pone.0225007 (PMC6863524; doi:10.1371/journal.pone.0225007)
Supplement: S2 Appendix — (DOCX) [file pone.0225007.s002.docx]

scafSLICR: a MATLAB-based Slicing Algorithm to Enable Fused Deposition Modeling 3D-Printing of Tissue Engineering Scaffolds with Heterogenous Porous Microarchitecture

Ethan Nyberg & Aine O'Sullivan, Warren Grayson

# Appendix B

Examples from the figures in manuscript.

# Example 1: Figure 3 Homogenous Cubes

clearvars

addpath('sub functions')

load('Shapes/rectangular.mat')

%Get GCODE Header and footer files

fileID = fopen(fullfile('gcode','header_abs.txt'),'r');

gstart = textscan(fileID,'%s','delimiter','\n');

gstart = string(gstart{1}); fclose(fileID);

fileID = fopen(fullfile('gcode','footer.txt'),'r');

gend = textscan(fileID,'%s','delimiter','\n');

gend = string(gend{1}); fclose(fileID);

% Read GCODE lines into output file

fileID = fopen(fullfile('Output','Figure3_Cubes.gcode'),'w');

formatSpec = '%s\n';

fprintf(fileID,formatSpec,gstart);

VoxelSize = 0.100;

PoreDiameter = [0.200 0.500 0.800 0.800 0.800] ;

Porosity = [0.280 0.250 0.280 0.450 0.620] ;

XPOS = 30; YPOS = 30; position = [XPOS YPOS];

for i = 1:length(PoreDiameter)

[glines, toolPaths, preview] = SlicrFn ( V, …

VoxelSize, …

PoreDiameter(i), …

Porosity(i), …

[XPOS YPOS],…

'Backtracking', false,…

'BedTemp', 110,…

'CreateBrim', true,…

'ExtruderTemp', 285,…

'ExtrusionMultiplier', 1.1,…

'FanHeight', 1,…

'FanPower', 95,…

'FilamentDiameter', 2.85,…

'LayerHeight', 0.200,…

'NozzleDiameter', 0.500,…

'PauseTime', 200,…

'PrintSpeed', 80,…

'SupportPoreDiam', 0.95…

);

glines(glines == '')=[]; %remove empty dimensions

fprintf(fileID,formatSpec,glines'); % Write to file

if XPOS > 230, XPOS = 30; YPOS = YPOS+40; else, XPOS = XPOS+40; end %if

end %for loop

%% Complete the GCODE as file

fprintf(fileID,formatSpec,gend);

fclose(fileID);

# Example 2: Figure 4 Hybrid Scaffolds

clearvars

addpath('sub functions')

VV = ones(200,200);

VV(1:100, ☺ = 2; VV = repmat(VV,1,1,100);

V = zeros(202, 202, 102); V(2:201, 2:201, 1:101) = VV;

%% Prepare the GCODE as file

fileID = fopen(fullfile('gcode','header_abs.txt'),'r');

gstart = textscan(fileID,'%s','delimiter','\n');

gstart = string(gstart{1}); fclose(fileID);

fileID = fopen(fullfile('gcode','footer.txt'),'r');

gend = textscan(fileID,'%s','delimiter','\n');

gend = string(gend{1}); fclose(fileID);

% Read GCODE lines into output file

fileID = fopen(fullfile('Output','Figure4_Hybrid.gcode'),'w');

formatSpec = '%s\n';

fprintf(fileID,formatSpec,gstart);

VoxelSize = 0.100;

PoreDiameter = [0.200 0.500; 0.500 0.800; 0.200 0.800; 0.800 0.800; 0.800 0.800; 0.800 0.800] ;

Porosity = [0.280 0.250; 0.250 0.280; 0.280 0.280; 0.280 0.450; 0.450 0.620; 0.280 0.620];

XPOS = 30; YPOS = 30; position = [XPOS YPOS];

for i = 1:length(PoreDiameter)

[glines, toolPaths, preview] = SlicrFn ( V, …

VoxelSize, …

PoreDiameter(i, ☺', …

Porosity(i, ☺, …

[XPOS YPOS],…

'Backtracking', false,…

'BedTemp', 110,…

'CreateBrim', true,…

'ExtruderTemp', 285,…

'ExtrusionMultiplier', 1.1,…

'FanHeight', 1,…

'FanPower', 95,…

'FilamentDiameter', 2.85,…

'LayerHeight', 0.200,…

'NozzleDiameter', 0.500,…

'PauseTime', 200,…

'PrintSpeed', 80,…

'SupportPoreDiam', 0.95…

);

glines(glines == '')=[]; %remove empty dimensions

fprintf(fileID,formatSpec,glines'); % Write to file

if XPOS > 230, XPOS = 30; YPOS = YPOS+40; else, XPOS = XPOS+40; end %if

end %for loop

%% Complete the GCODE as file

fprintf(fileID,formatSpec,gend);

fclose(fileID);

# Example 3: Figure 5 Gradients in Cubes

Choose the case to create the 3D pattern.

**Graded Z**

VV = ones(200,200,200);

num = 10; width = 200/num; x1=0; x2=width;

for i=1:num

VV(:,:, x1+1:x2) = i;

x1=x2; x2=x2+width;

end

V=zeros(202,202,202);

V(2:201, 2:201,1:201) = VV;

**Graded along X**

VV = ones(200,200,200);

num = 10; width = 200/num; x1=0; x2=width;

for i=1:num

VV(:,x1+1:x2,☺ = i;

x1=x2; x2=x2+width;

end

V=zeros(202,202,202);

V(2:201, 2:201,1:201) = VV;

**Graded along XY**

VV = ones(200,200);

num = 10; width = 200/num; x1=0; x2=200;

for i = 1☹num/2)

VV(x1+1:x2,x1+1:x2) = i;

x1=x1+width; x2=x2-width;

end

VV = repmat(VV, [1, 1, 200]);

V=zeros(202,202,202);

V(2:201, 2:201,1:201) = VV;

**Graded along XYZ**

VV = ones(200,200, 200);

num = 10; width = 200/num; x1=0; x2=200;

for i = 1☹num/2)

VV(x1+1:x2,x1+1:x2, x1+1:x2) = i;

x1=x1+width; x2=x2-width;

end

V=zeros(202,202,202);

V(2:201, 2:201,1:201) = VV;

addpath('sub functions')

VoxelSize = 0.200;

PoreDiameter = ([ 0.2 0.3 0.5 0.6 0.8 0.8 0.6 0.5 0.3 0.2]');

Porosity = ([0.28 0.26 0.5 0.56 0.62 0.62 0.56 0.5 0.26 0.28]);

position = [30 30];

[glines, toolPaths, preview] = SlicrFn ( V, …

VoxelSize, …

PoreDiameter, …

Porosity, …

position,…

'Backtracking', false,…

'BedTemp', 110,…

'CreateBrim', true,…

'ExtruderTemp', 285,…

'ExtrusionMultiplier', 1.1,…

'FanHeight', 1,…

'FanPower', 95,…

'FilamentDiameter', 2.85,…

'LayerHeight', 0.200,…

'NozzleDiameter', 0.500,…

'PauseTime', 200,…

'PrintSpeed', 80,…

'SupportPoreDiam', 0.95);

% Get GCODE Header and footer files

fileID = fopen(fullfile('gcode','header_abs.txt'),'r');

gstart = textscan(fileID,'%s','delimiter','\n');

gstart = string(gstart{1});

fclose(fileID);

fileID = fopen(fullfile('gcode','footer.txt'),'r');

gend = textscan(fileID,'%s','delimiter','\n');

gend = string(gend{1});

fclose(fileID);

% Read GCODE lines into output file

fileID = fopen(fullfile('output','Figure5-subpart.gcode'),'w');

formatSpec = '%s\n';

fprintf(fileID,formatSpec,gstart);

glines(glines == '')=[]; %remove empty dimensions

fprintf(fileID,formatSpec,glines');

fprintf(fileID,formatSpec,gend);

fclose(fileID);

# Example 4: Figure 6 Graded Zygoma

clearvars

addpath('sub functions')

load('Shapes/zygoma-5regions.mat')

VoxelSize = 0.600;

PoreDiameter = ([ 0.2 0.3 0.5 0.7 0.9]');

Porosity = ([0.3 0.3 0.5 0.5 0.8]);

position = [30 30];

[glines, toolPaths, preview] = SlicrFn ( V, ...

VoxelSize, ...

PoreDiameter, ...

Porosity, ...

position,...

'Backtracking', false,...

'BedTemp', 110,...

'CreateBrim', true,...

'ExtruderTemp', 285,...

'ExtrusionMultiplier', 1.1,...

'FanHeight', 1,...

'FanPower', 95,...

'FilamentDiameter', 2.85,...

'LayerHeight', 0.200,...

'NozzleDiameter', 0.500,...

'PauseTime', 200,...

'PrintSpeed', 80,...

'SupportPoreDiam', 0.95...

);

%% Complete the GCODE as file

% Get GCODE Header and footer files

fileID = fopen(fullfile('gcode','header_abs.txt'),'r');

gstart = textscan(fileID,'%s','delimiter','\n');

gstart = string(gstart{1});

fclose(fileID);

fileID = fopen(fullfile('gcode','footer.txt'),'r');

gend = textscan(fileID,'%s','delimiter','\n');

gend = string(gend{1});

fclose(fileID);

% Read GCODE lines into output file

fileID = fopen(fullfile('output','SlicedCode-PorcineZygoma.gcode'),'w');

formatSpec = '%s\n';

fprintf(fileID,formatSpec,gstart);

glines(glines == '')=[];

fprintf(fileID,formatSpec,glines');

fprintf(fileID,formatSpec,gend);

fclose(fileID);

# Example 5: Figure 6 Orbital Bone

clearvars

addpath('sub functions')

% Import STL

file = 'Shapes\MassiveOrbitalScaffold.stl';

% Get the dimensions of the STL in millimeters.

[stlcoords] = READ_stl(file);

xmax = max(max( squeeze( stlcoords(:,1,:) ) ));

ymax = max(max( squeeze( stlcoords(:,2,:) ) ));

zmax = max(max( squeeze( stlcoords(:,3,:) ) ));

xmin = min(min( squeeze( stlcoords(:,1,:) ) ));

ymin = min(min( squeeze( stlcoords(:,2,:) ) ));

zmin = min(min( squeeze( stlcoords(:,3,:) ) ));

% Convert to voxel matrix where one voxel is 1mm

V = double(VOXELISE(round((xmax-xmin)*1),...

round((ymax-ymin)*1),...

round((zmax-zmin)*1),...

fullfile(path,file),...

'xyz'));

%% Weighted Average

% V is the VOI, one voxel is 1mm

[a,b,c] = size(V);

d = size(V(:),1);

output = V*0;

for i=1:d

if V(i)==0, continue, end

[x,y,z] = ind2sub([a,b,c],i);

top = x + 4; if top>a, top = a; end

bottom = x - 4; if bottom<1, bottom = 1; end

left = y - 4; if left<1, left = 1; end

right = y + 4; if right>b, right = b; end

in = z - 4; if in<1, in =1; end

out = z + 4; if out>c, out = c; end

V_small = V(bottom:top, left:right, in:out);

output(i) = sum(sum(sum(V_small)));

end

figure, histogram(output(output>0));

figure, plot_3d(output, 10, .5)

%% Smooth the weighted average twice

avg_out = output*0;

for i=1:d

if V(i)==0, continue, end

[x,y,z] = ind2sub([a,b,c],i);

top = x + 8; if top>a, top = a; end

bottom = x - 8; if bottom<1, bottom = 1; end

left = y - 8; if left<1, left = 1; end

right = y + 8; if right>b, right = b; end

in = z - 8; if in<1, in =1; end

out = z + 8; if out>c, out = c; end

V_small = output(bottom:top, left:right, in:out);

V_small = V_small(:);

V_small(V_small==0)=[];

avg_out(i) = round(mean(V_small(:)));

end

figure, histogram(avg_out(avg_out>0));

figure, plot_3d(avg_out, 10, .5)

avg_out1 = output*0;

for i=1:d

if V(i)==0, continue, end

[x,y,z] = ind2sub([a,b,c],i);

top = x + 8; if top>a, top = a; end

bottom = x - 8; if bottom<1, bottom = 1; end

left = y - 8; if left<1, left = 1; end

right = y + 8; if right>b, right = b; end

in = z - 8; if in<1, in =1; end

out = z + 8; if out>c, out = c; end

V_small = avg_out(bottom:top, left:right, in:out);

V_small = V_small(:);

V_small(V_small==0)=[];

avg_out1(i) = round(mean(V_small(:)));

end

figure, plot_3d(avg_out1, 10, .5)

%% Bin smoothed range into 4 levels

avg_sort = sort(avg_out(avg_out>0));

fifths = round(size(avg_sort, 1) / 5);

figure, histogram(avg_out1(avg_out1>0)); hold on

line([avg_sort(fifths), avg_sort(fifths)], ylim, 'LineWidth', 2, 'Color', 'r');

line([avg_sort(fifths*2), avg_sort(fifths*2)], ylim, 'LineWidth', 2, 'Color', 'r');

line([avg_sort(fifths*3), avg_sort(fifths*3)], ylim, 'LineWidth', 2, 'Color', 'r');

line([avg_sort(fifths*4), avg_sort(fifths*4)], ylim, 'LineWidth', 2, 'Color', 'r');

bin_out = avg_out;

bin_out(0 < avg_out & avg_out < avg_sort(fifths)) = 1;

bin_out(avg_sort(fifths) <= avg_out & avg_out < avg_sort(fifths*2)) = 2;

bin_out(avg_sort(fifths*2) <= avg_out & avg_out < avg_sort(fifths*3)) = 3;

bin_out(avg_sort(fifths*3) <= avg_out & avg_out < avg_sort(fifths*4)) = 4;

bin_out(avg_sort(fifths*4) <= avg_out & avg_out <= avg_sort(end)) = 5;

figure, histogram(bin_out(bin_out>0));

figure, plot_3d(bin_out, .5, .5)

# Example 6: Figure 6 Hemi-Mandible

clearvars

addpath('sub functions')

% Import STL

file = 'Shapes/HemiMandible_001.stl';

% Get the dimensions of the STL in millimeters.

[stlcoords] = READ_stl(file);

xmax = max(max( squeeze( stlcoords(:,1,:) ) ));

ymax = max(max( squeeze( stlcoords(:,2,:) ) ));

zmax = max(max( squeeze( stlcoords(:,3,:) ) ));

xmin = min(min( squeeze( stlcoords(:,1,:) ) ));

ymin = min(min( squeeze( stlcoords(:,2,:) ) ));

zmin = min(min( squeeze( stlcoords(:,3,:) ) ));

% Convert to voxel matrix where one voxel is 1mm

V = double(VOXELISE(round((xmax-xmin)*1),...

round((ymax-ymin)*1),...

round((zmax-zmin)*1),...

fullfile(file),...

'xyz'));

V = flipud(permute(V,[3 1 2]));

%% Rotate

h(1) = figure;

p1= plot_3d(V, 0.5, 0.5);

title('Input Shape')

xlabel('X')

ylabel('Y')

zlabel('Z')

%% Rotation about each axes

tx = 0;

ty = 0;

tz = 0;

tx = pi*1.1 + pi/5;

ty = pi/6 +pi/2; %about Z

Rx = [1 0 0 0; ...

0 cos(tx) -sin(tx) 0; ...

0 sin(tx) cos(tx) 0;...

0 0 0 1];

Ry = [cos(ty) 0 sin(ty) 0; ...

0 1 0 0;...

-sin(ty) 0 cos(ty) 0;...

0 0 0 1];

Rz = [cos(tz) -sin(tz) 0 0; ...

sin(tz) cos(tz) 0 0;...

0 0 1 0;...

0 0 0 1];

% Complete Rotation Transformation

t = Rx * Ry *Rz ;

tform = affine3d(t);

% Transform Image

V_rot = imwarp(V,tform, 'nearest');

% Crop V_rot

Ibw = V_rot~=0;

I_xy = sum(Ibw,3);

I_x = squeeze(sum(I_xy,1));

I_y = squeeze(sum(I_xy,2));

I_xz = squeeze(sum(Ibw, 2));

I_z = squeeze(sum(I_xz, 1));

x1 = find(I_x, 1, 'first');

x2 = find(I_x, 1, 'last');

y1 = find(I_y, 1, 'first');

y2 = find(I_y, 1, 'last');

z1 = find(I_z, 1, 'first');

z2 = find(I_z, 1, 'last');

V_rot = V_rot(y1:y2, x1:x2,z1:z2);

h(2) = figure;

p(2) = plot_3d(V_rot, 0.1, 0.5);

title('Out Shape')

xlabel('X')

ylabel('Y')

zlabel('Z')

% Depth-based Regions

% V_rot is the VOI, one voxel is 1mm

% Want to make shells from the outside --> inside

% that are 3mm in thickness

V = V_rot;

output = V*0;

V_shell = V>0;

finished = false;

i = 1; j = 1;

while ~finished

shell = bwmorph3(V_shell, 'remove');

if ~any(shell(:))

finished=true;

continue

end

output = output + double(shell*i);

V_shell = V_shell-shell;

j = j+1;

if j>3

i = i+1;

j=1;

end %if

end %while not finished

%% Slice Scaffold

output(output>3)=3;

V = rot90(output);

VoxelSize = 1;

PoreDiameter = [ 0.8 0.4 0.1 ]';

Porosity = [ 0.6154 0.2857 0.0099 ];

% Porosity and PoreDiameter must be the same length as the number of

% non-zero unique values in V, and are ordered respectively to the

% non-zero output of unique(V).

position = [15 15];

[glines, toolPaths, preview] = SlicrFn ( V, ...

VoxelSize, ...

PoreDiameter, ...

Porosity, ...

position,...

'Backtracking', false,...

'BedTemp', 110,...

'CreateBrim', true,...

'ExtruderTemp', 285,...

'ExtrusionMultiplier', 1.1,...

'FanHeight', 1,...

'FanPower', 95,...

'FilamentDiameter', 2.85,...

'LayerHeight', 0.200,...

'NozzleDiameter', 0.500,...

'PauseTime', 200,...

'PrintSpeed', 600,...

'SupportPoreDiam', 0.95...

);

% Complete the GCODE as file

% Get GCODE Header and footer files

fileID = fopen(fullfile('gcode','header_abs.txt'),'r');

gstart = textscan(fileID,'%s','delimiter','\n');

gstart = string(gstart{1});

fclose(fileID);

fileID = fopen(fullfile('gcode','footer.txt'),'r');

gend = textscan(fileID,'%s','delimiter','\n');

gend = string(gend{1});

fclose(fileID);

% Read GCODE lines into output file

fileID = fopen(fullfile('gcode','SlicedCode.gcode'),'w');

formatSpec = '%s\n';

fprintf(fileID,formatSpec,gstart);

glines(glines == '')=[]; %remove empty dimensions

fprintf(fileID,formatSpec,glines');

fprintf(fileID,formatSpec,gend);

fclose(fileID);

# Example 7: Throughput Testing Case

Cylinders, with pore sizes 0.200mm to 0.800 in 0.2mm steps and print Speed from 100mm/min to 500mm/min


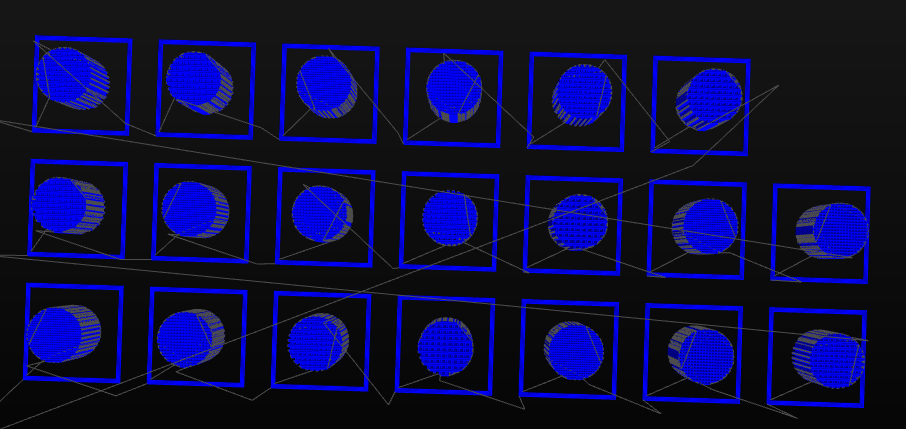


clearvars

addpath('sub functions')

%% Make a Cylinder Shape

ix=200;iy=200;

r=85;

cx=100;cy=100;

he = ix;

[x,y]=meshgrid(-(cx-1):(ix-cx),-(cy-1):(iy-cy));

c_mask=((x.^2+y.^2)<=r^2);

V = repmat(c_mask, [1 1 he]);

V(:,:, he-4:he)=0;

V = double(V);

%% Prepare the GCODE as file

fileID = fopen(fullfile('gcode','header_abs.txt'),'r');

gstart = textscan(fileID,'%s','delimiter','\n');

gstart = string(gstart{1});

fclose(fileID);

fileID = fopen(fullfile('gcode','footer.txt'),'r');

gend = textscan(fileID,'%s','delimiter','\n');

gend = string(gend{1});

fclose(fileID);

% Read GCODE lines into output file

fileID = fopen(fullfile('Output','Figure9_Throughput.gcode'),'w');

formatSpec = '%s\n';

fprintf(fileID,formatSpec,gstart);

%% Slicing

VoxelSize = 0.100;

PoreDiameter = [0.200 0.400 0.600 0.800] ;

Porosity = [0.5 0.5 0.5 0.5] ;

XPOS = 30; YPOS = 30;

position = [XPOS YPOS];

printSpeeds = [20 40 80 100 110];

for j = 1:length(printSpeeds)

for i = 1:length(PoreDiameter)

[glines, toolPaths, preview] = SlicrFn ( V, ...

VoxelSize, ...

PoreDiameter(i)', ...

Porosity(i), ...

[XPOS YPOS],...

'Backtracking', false,...

'BedTemp', 110,...

'CreateBrim', true,...

'ExtruderTemp', 285,...

'ExtrusionMultiplier', 1.1,...

'FanHeight', 1,...

'FanPower', 95,...

'FilamentDiameter', 2.85,...

'LayerHeight', 0.200,...

'NozzleDiameter', 0.500,...

'PauseTime', 200,...

'PrintSpeed', printSpeeds(j),...

'SupportPoreDiam', 0.95...

);

glines(glines == '')=[]; %remove empty dimensions

fprintf(fileID,formatSpec,glines'); % Write to file

if XPOS > 230, XPOS = 30; YPOS = YPOS+40; else, XPOS = XPOS+40; end %if

end %for pore diameter loop

end %for print speeds loop

fprintf(fileID,formatSpec,gend);

fclose(fileID);
